# Ancestor of land plants acquired the DNA-3-methyladenine glycosylase (*MAG*) gene from bacteria through horizontal gene transfer

**DOI:** 10.1038/s41598-017-05066-w

**Published:** 2017-08-24

**Authors:** Huimin Fang, Liexiang Huangfu, Rujia Chen, Pengcheng Li, Shuhui Xu, Enying Zhang, Wei Cao, Li Liu, Youli Yao, Guohua Liang, Chenwu Xu, Yong Zhou, Zefeng Yang

**Affiliations:** 1 Jiangsu Key Laboratory of Crop Genetics and Physiology/Co-Innovation Center for Modern Production Technology of Grain Crops, Key Laboratory of Plant Functional Genomics of the Ministry of Education, Yangzhou University, Yangzhou, 225009 China; 2 College of Agronomy and Plant Protection, Qingdao Agricultural University, 266109 Qingdao, China

## Abstract

The origin and evolution of land plants was an important event in the history of life and initiated the establishment of modern terrestrial ecosystems. From water to terrestrial environments, plants needed to overcome the enhanced ultraviolet (UV) radiation and many other DNA-damaging agents. Evolving new genes with the function of DNA repair is critical for the origin and radiation of land plants. In bacteria, the DNA-3-methyladenine glycosylase (MAG) recognizes of a variety of base lesions and initiates the process of the base excision repair for damaged DNA. The homologs of *MAG* gene are present in all major lineages of streptophytes, and both the phylogenic and sequence similarity analyses revealed that green plant *MAG* gene originated through an ancient horizontal gene transfer (HGT) event from bacteria. Experimental evidence demonstrated that the expression of the maize *ZmMAG* gene was induced by UV and zeocin, both of which are known as DNA-damaging agents. Further investigation revealed that Streptophyta *MAG* genes had undergone positive selection during the initial evolutionary period in the ancestor of land plants. Our findings demonstrated that the ancient HGT of *MAG* to the ancestor of land plants probably played an important role in preadaptation to DNA-damaging agents in terrestrial environments.

## Introduction

The origin of land plants (Embryophytes) is one of the most important events in the evolution of life on earth and was the key step in the process of developing modern terrestrial ecosystems. Numerous lines of evidence have revealed that land plants evolved from water-based green algae^1–3^. From water to terrestrial environments, plants have enjoyed the advantages of the land, such as brighter sunlight, more carbon dioxide in the atmosphere and more plentiful mineral nutrients in the soil. On the other hand, land plants have had to overcome challenges in terrestrial environments, including less water in land and no support against gravity. Some new structures, including cuticles, vascular tissue, roots and leaves, have evolved to adapt these abominable environments on land^4, 5^. Due to their sessile nature, land plants are continuously exposed to DNA-damaging agents, including UVB, ozone, desiccation, rehydration, salinity, low and high temperature, and air and soil pollutants including metals-metalloids. In addition, some other environmental stressors, including chemical mutagens, ionizing radiations, alkylating agents, aromatic compounds and microbial toxins, can also damage the DNA in land plants^6, 7^. Plants use light energy from the sun for photosynthesis to make their chemicals. However, sunlight is the greatest source of UV radiations. The photosynthetic characteristics and sessile nature of land plants make it unpreventable to avoid UV radiation. As a part of ultraviolet radiation, UVB (280–315 nm) is directly absorbed by DNA and induces various DNA lesions such as cyclobutane pyrimidine dimers (CPDs), 6-4 pyrimidine-pyrimidone photoproducts (6-4 PPs), and other minor types of DNA damage. Every pyrimidine dimer blocks transcription and replication and is sufficient to completely eliminate expression of a transcriptional unit^6, 8–10^. Therefore, effective mechanisms of DNA repair are essential for adaptation to terrestrial environments, and hence to ensure the stability of the genome for plants^11^.

DNA glycosylases catalyze the first step of base excision repair (BER) pathway, the mechanism by which damaged bases in DNA are removed and replaced^12^. These enzymes remove the damaged nitrogenous base while leaving the sugar-phosphate backbone intact, creating an apurinic/apyrimidinic site (AP site)^13, 14^. DNA-3-methyladenine glycosylase (MAG), which is also known as 3-alkyladenine DNA glycosylase (AAG) or N-methylpurine DNA glycosylase (MPG), is thought to be involved in the recognition of a variety of base lesions, including alkylated and deaminated purines and to initiate their repair *via* the BER pathway^15^. The broad substrate recognition by MAG serves to provide resistance to a wide variety of DNA damaging agents^16^. Although nearly all MAGs share the ability to rescue 3-methyladenine DNA glycosylase-deficient *Escherichia coli* from death induced by the alkylating agent methylmethane sulfonate (MMS), the MAGs can be divided into several subfamilies that act on a wide variety of damaged DNA bases^16^. However, some MAGs were found to remove normal bases from the genome, suggesting that they may have detrimental consequences under certain conditions^17–19^.

In land plants, the gene encoding MAG was first isolated in *Arabidopsis thaliana*, and this gene complements the MMS-sensitive phenotype of an *E. coli* double mutant deficient in 3-methyladenine glycosylases^20, 21^. The *MAG* genes have also been detected in some other higher plants, including maize^22, 23^, wheat^24^, grape^25^ and *Brachypodium distachyon* ^26^. These results suggested that the homologs of the *Arabidopsis MAG* gene might be present in a wide range of land plants, and the ubiquity of this gene in land plants suggests that its functions include a wide range of selectivity. Here, we provide information that helps answer some questions regarding the evolution of land plant *MAG* gene by performing an extensive search of its homologs in current sequence databases and by analyzing their phylogeny.

## Results

### *MAG* genes are widely present in streptophytes

Comprehensive BLAST searches revealed that *MAG* genes are widely present in various streptophytes, from charophyte algae to bryophytes, pteridophytes and seed plants (Supplementary Table S1). However, no *MAG* homologs were detected in any genomes of chlorophyte algae. In the genomes of the moss *Physcomitrella patens* and the gymnosperm *Picea abies* that have been fully sequenced, no *MAG* homologs were found. In addition, no EST hits of *MAG* genes were observed in these two species. However, we noticed that another moss genome, the *Sphagnum fallax*, contained one *MAG* gene. Some EST hits in gymnosperm *P. glauca* were matched to the *MAG* homologs. These results suggested that the moss *P. patens* and the gymnosperm *P. abies* have lost the *MAG* gene during evolution. In addition to the genome of *Klebsormidium flaccidum*, another charophyte alga, *Penium margaritaceum*, possesses ESTs which show high similarity with the *MAG* genes, suggesting that this gene might be present widely in charophyte algae.

**Figure 1:**
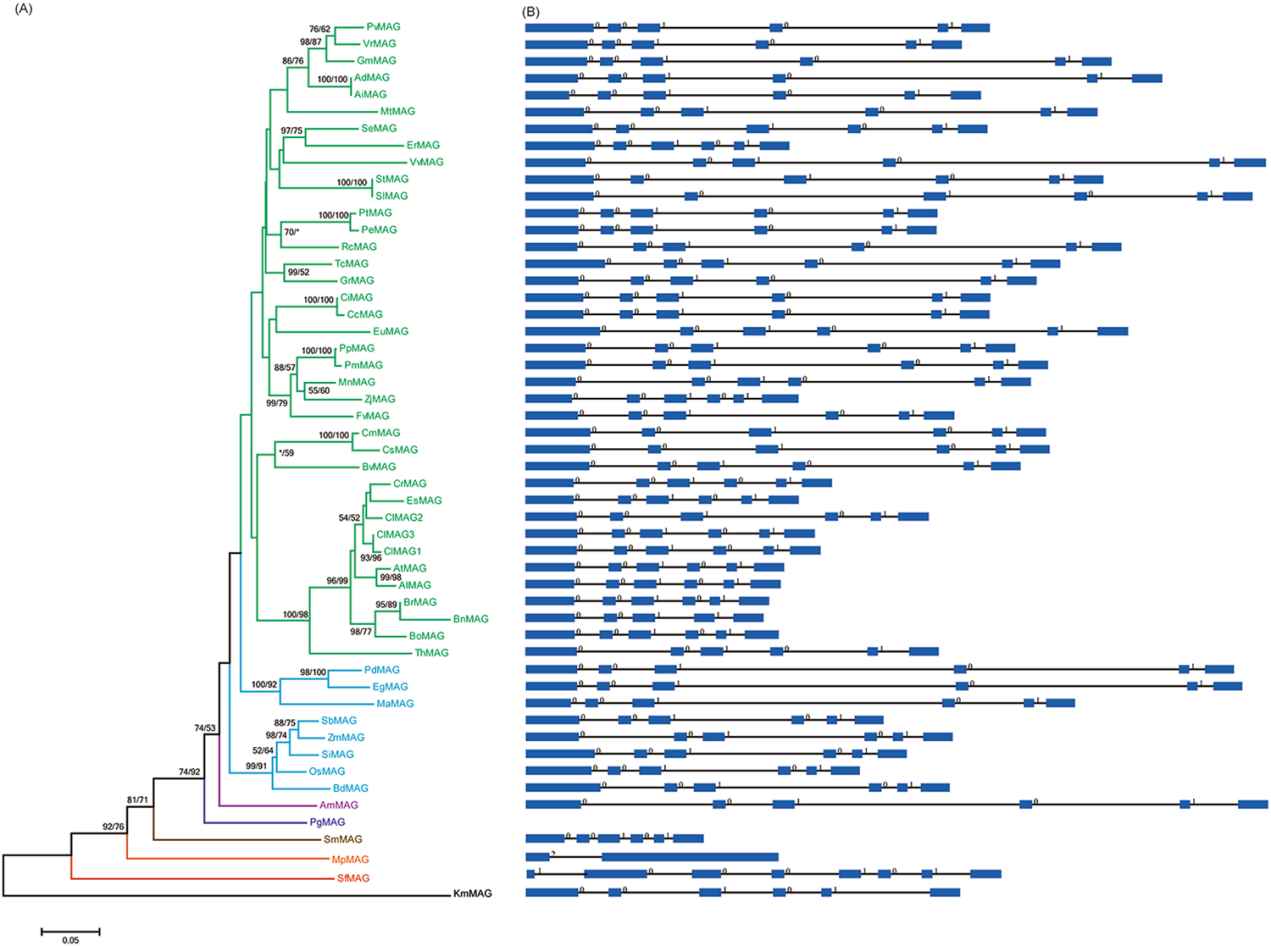
Phylogenetic tree of green plant DNA-3-methyladenine glycosylase (*MAG*) genes and their exon/intron structures. The numbers above the branches represent the bootstrap values for the maximum likelihood and distance analyses, respectively. The asterisks indicate values <50%. The exons are indicated by boxes, and introns are indicated by lines. The number above an intron indicates the phase.

To investigate the evolution of *MAG* genes in green plants, we examined genomes representing the main lineages of streptophytes, including the charophyte alga *K. flaccidum*, the moss *S. fallax*, the liverwort *Marchantia polymorpha*, the lycophyte *Selaginella moellendorffii*, the gymnosperm *P. glauca*, the basal angiosperm *Amborella trichopoda*, and eight monocot and 36 dicot angiosperms (Supplementary Table S1 and Fig. 1). All these genomes contain only one *MAG* gene except for the dicot *Camelina sativa*, which possesses three *MAG* genes. In the phylogeny, all *C. sativa MAG* paralogs were located at the termini of branches, suggesting that these paralogs were formed through recent duplication events after split with other species. Further evidence revealed that these three paralogous genes resulted from segmental duplication because they are located in different chromosomes and there were highly conserved genes within the flanking regions of these paralogous *MAG* genes. Nearly all of the selected *MAG* genes contained five introns in their coding regions, and their positions and phases showed characteristics of conservation. This investigation suggested the plant *MAG* genes have highly similar gene structure and the main features of the *MAG* gene was established before the origin of land plants. However, we did notice that two of the plant *MAG* genes showed different exon/intron structure than others, the moss *S. fallax* gene *SfMAG* and the liverwort *M. polymorpha* gene *MpMAG*. Through comparing its sequence with others, we conclude that the *SfMAG* gene had acquired 2 introns in its 5′ region. The gene *MpMAG* only contained one intron in coding region, and its position and phase of this gene is different from other plant *MAG* genes, suggesting that it might be formed through reconstruction of exon/intron structure (Fig. 1).

### Streptophyta *MAG* gene was acquired through an ancient horizontal gene transfer event

**Figure 2:**
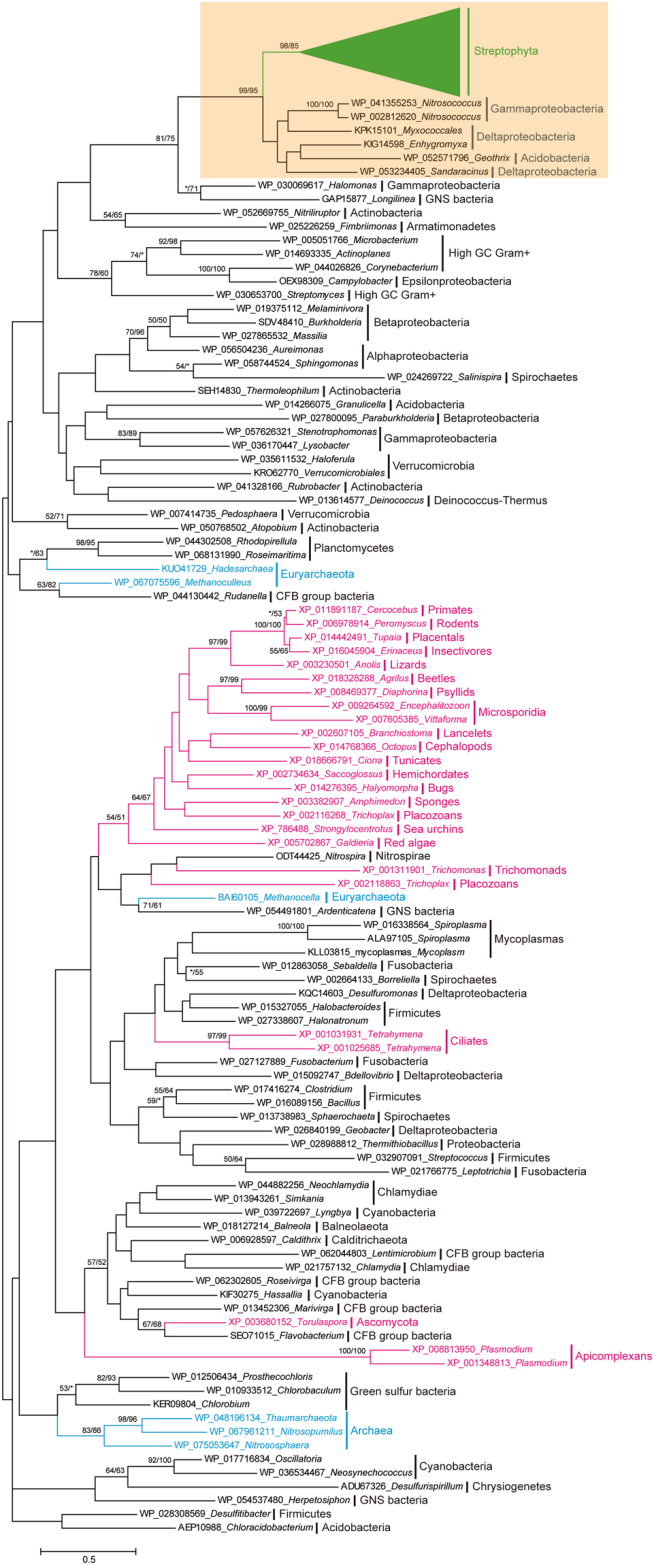
Phylogenetic analyses of MAG proteins. The numbers above the branches are the bootstrap values for maximum likelihood and distance analyses. Asterisks indicate values lower than 50%. Brown yellow shading indicates lineages in which land plant *MAG* genes evolved. Green, red, blue and black branches indicate genes from streptophyta, eukaryota, archaea and bacteria, respectively. All sequences were obtained from NCBI, except for those in streptophyta, and the locus number in NCBI and genus is given for each protein.

Similarity searches also revealed that homologs of Streptophyta *MAG* genes are widely present in many cellular organisms, including red algae, fungi, ciliates, trichomonads, apicomplexans, metazoan, bacteria and Archaea. The universality of their distribution in bacteria also suggested that this gene first emerged in bacteria. In addition, we noticed that the green plant MAG proteins showed higher similarity with homologs in bacteria than those in other eukaryotes. To determine the origin of the Streptophyta *MAG* genes, we selected representative homologs from each taxonomic group of cellular organisms in the *nr* database to build a phylogenetic tree (Fig. 2). Each of the proteins selected for phylogenetic analysis possessed a Pur_DNA_glyco domain (PF02245). In the phylogenetic tree, all of the Streptophyta *MAG* formed a single clade with high bootstrap supports. The monophyly of the Streptophyta *MAG* genes strongly suggests that they have a single origin and are derived from a unique gene that was present in the ancestor of the Streptophyta. Although some other eukaryotic genomes contained the genes encoding MAGs, they did not follow the same clade with those in green plants, suggesting that the Streptophyta *MAG* genes have a different origin pattern than those in other eukaryotes. In addition, we noticed that the Streptophyta *MAG* genes fell within the branch of bacterial genes showing high bootstrap support values in both maximum likelihood and distance analyses. The bacterial genes in this branch come from species of gammaproteobacteria, deltaproteobacteria, and acidobacteria. Furthermore, we observed that the Streptophyta MAG proteins showed the highest similarity to those of bacteria in this clade; in particular, at least three motifs in the C-terminus are only conserved in green plant and these bacterial MAGs (Supplementary Fig. 1). These findings illustrate that the ancestor of streptophytes had possibly acquired the *MAG* gene through a single ancient HGT event from bacteria prior to the origin of land plants.

### Positive selection facilitated the evolution of the *MAG* genes in streptophytes

To test the selective constraints on the evolution of *MAG* genes, we used the site-specific model to detect positively selected sites for the sampled Streptophyta *MAG* genes and six bacterial genes following the same branch as plants. The maximum likelihood estimates of the *d*_*N*_/*d*_*s*_ values under M0 model for plant and bacterial groups were 0.0740 and 0.0486 (Supplementary Table S2), respectively. These two estimates are close to zero, suggesting that purifying selection was the predominant force for the evolution of the *MAG* genes both in streptophytes and selected bacterial lineages. However, the log-likelihood differences between models M3 and M0 were statistically significant for both lineages, indicating that the selective constraint level varied across amino acid positions. To determine whether positive selection promoted the divergence of the *MAG* genes in both lineages, two likelihood ratio tests were employed to compare the data fit to models M2a *vs* M1a and M8 *vs* M7. None of the LRTs were statistically significant, and no amino acid site was found to be influenced by positive selection during the evolution of *MAG* genes either in streptophytes or in bacteria (Supplementary Table S2). These results suggested that purifying selection was the predominant force in the evolution of both the Streptophyta and bacterial *MAG* genes, and no positive selection signature was found during their evolution either in these two lineages.

**Figure 3:**
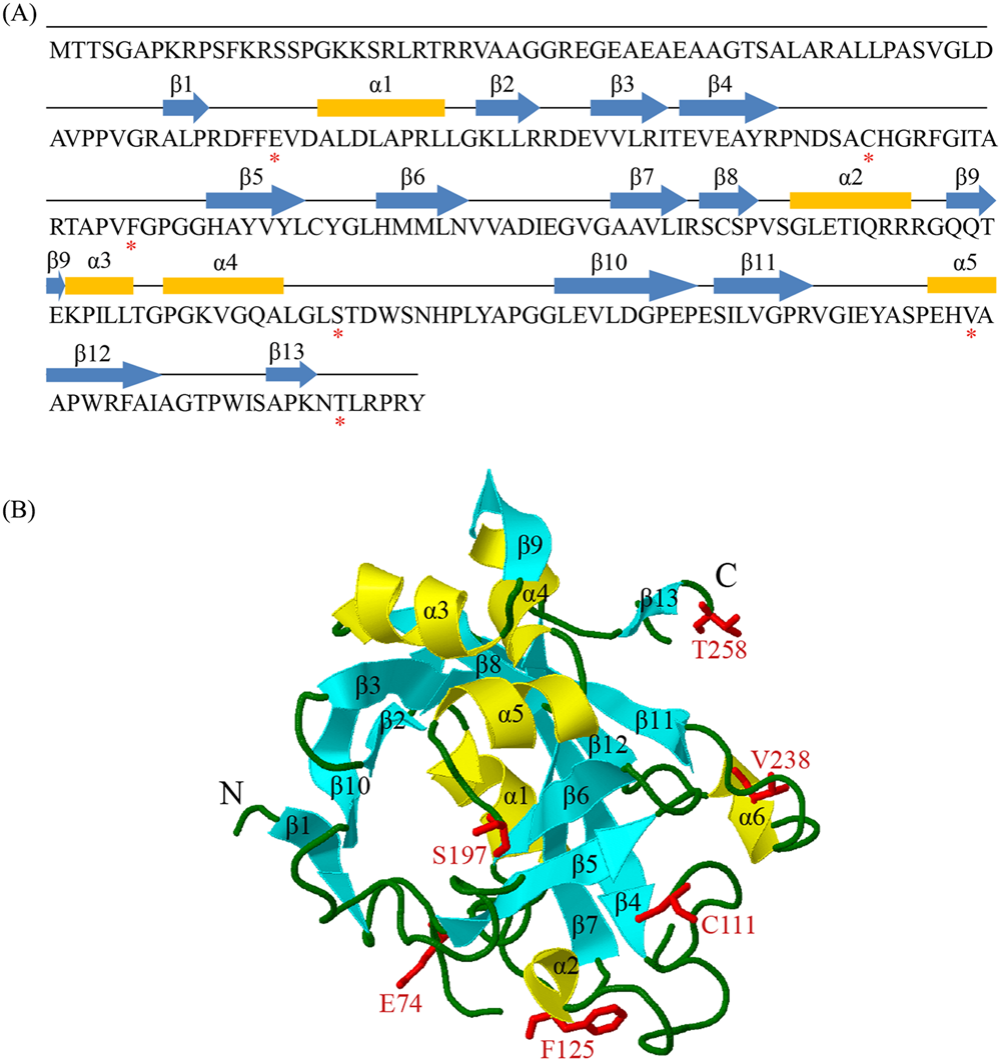
Putative positive selection sites of ZmMAG. (**A**) Secondary structure of ZmMAG. The positive selection residues are indicated with asterisk. (**B**) 3D structure of ZmMAG. The positive selection residues are marked and are colored in different colors. The structure was predicted using the tool of SWISS-MODEL and was displayed by the software Geneious.

To detect whether streptophytes are characterized by a different pattern of molecular evolution for the *MAG* genes compared with that in bacteria, an improved branch-site model^27^ was used to identify the positively selected sites in Streptophyta genes. A new phylogenetic tree was reconstructed using the green plant and bacterial MAG proteins, and the branch of Streptophyta *MAG* genes was classified as the foreground branch, while those in bacteria as the background branches. In this analysis, only the bacterial genes following to the same branch of plants were selected. We found that the model that permitted a class of positively selected codons with *d*_*N*_/*d*_*s*_ > 1 for the Streptophyta branch had a significantly better fit to the data than the model in which this class of codon was restricted to *d*_*N*_/*d*_*s*_ = 1 (Table 1). The method of Bayes empirical Bayes^28^ was further employed to determine the positively selected sites and their posterior probabilities, and 6 codons were found to have a 95% posterior probability of positive selection (Supplementary Fig. 2). The predicted three-dimensional structure of the maize ZmMAG protein possessed the thirteen β-strands (β1-β13) and six regular α-helices (α1-α6). We noticed that only one positively selected sites (V238) is located in the region of helix, while all the other five sites are located in regions connecting the α-helices and β-strands (Fig. 3). These observations suggest that positive selection pressure on these residues might have changed the protein structure, thus accelerated functional divergence. The most reasonable explanation for these results is that the *MAG* gene underwent adaptive evolution in the streptophyte ancestor during a short period of time after the HGT event, although the dominant force for the evolution in streptophytes of these codons is purifying selection.

**Table 1:** Parameters of the branch-site models used for the detection of positive selection.

Model	ln*L*	Parameters
	−13597.9228	*p*_0_ = 0.8510, *p*_1_ = 0.0485, *p*_2*a*_ = 0.0950, *p*_2b_ = 0.0054
		Background: *ω*_0_ = 0.0604, *ω*_1_ = 1.0000, *ω*_2*a*_ = 0.0604, *ω*_2*b*_ = 1.0000
		Foreground: *ω*_0_ = 0.0604, *ω*_1_ = 1.0000, *ω*_2*a*_ = 1.0000, *ω*_2*b*_ = 1.0000
		*p*_0_ = 0.8327, *p*_1_ = 0.0482, *p*_2*a*_ = 0.1126, *p*_2*b*_ = 0.0065
alternative	−13593.2870^**^	Background: *ω*_0_ = 0.0609, *ω*_1_ = 1.0000, *ω*_2*a*_ = 0.0609, *ω*_2*b*_ = 1.0000
		Foreground: *ω*_0_ = 0.0609, *ω*_1_ = 1.0000, *ω*_2*a*_ = 249.5960, *ω*_2*b*_ = 249.5960

**P < 0.01.

### Expression of the maize *MAG* gene is regulated by DNA-damaging agents

**Figure 4:**
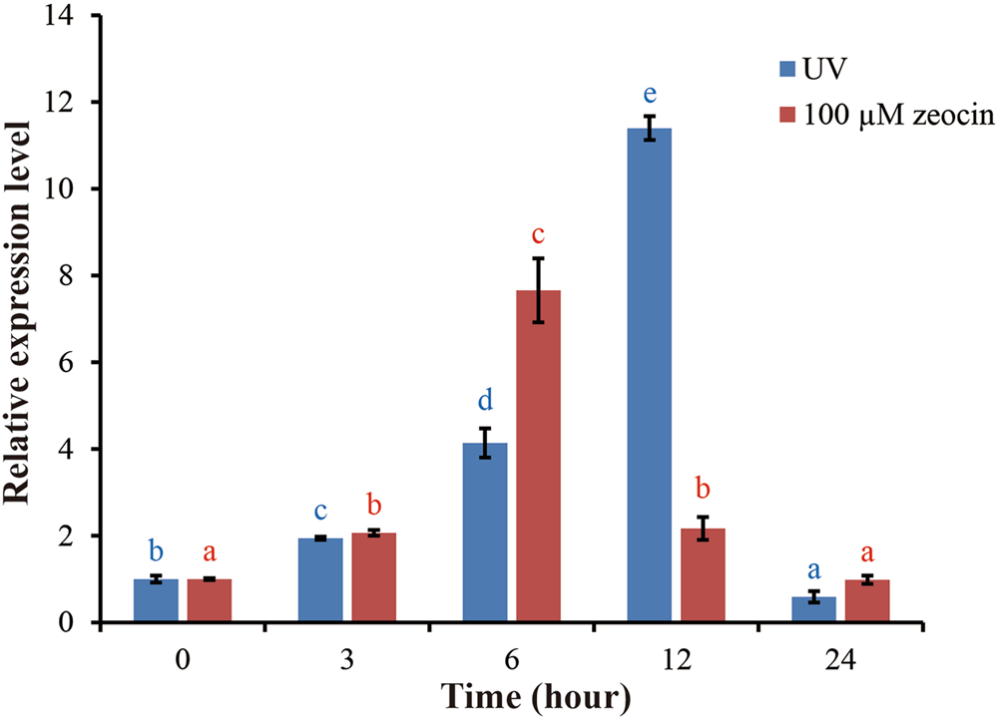
Real-time PCR analysis for the expression of *ZmMAG* in response to UV and zeocin treatments. Two-week-old maize seedlings grown in soil were collected for gene expression analysis of different time intervals. Total RNA was prepared from 2-week-old seedlings of wild-type maize after UV and zeocin treatments, respectively, and then reverse-transcribed. The resultant cDNAs were used as templates for real-time PCR analysis and *ZmActin* was used as an internal control. Real-time PCR was performed with *ZmMAG* specific primers and *ZmActin* specific primers. Data represent means and standard deviation of three replicates. Different letters following mean values indicate significant difference (*P* < 0.05, Tukey’s honestly significant difference test).

The gene expression pattern can provide valuable information on the function of a protein. In this study, we examined the expression pattern of the maize (*Zea mays* L.) *MAG* gene in response to DNA-damaging agents. First, we detected the expression level of this gene following treatment with UV light (Fig. 4). The *ZmMAG* transcripts gradually increased in the maize seedlings within the first 3 h after exposure to UV treatment, and peaked with the highest expression level at 12 h. Second, we noticed that the treatment of zeocin also up-regulated the expression of *ZmMAG* gene, and the level of expression reached the highest level at 6 h. However, the expression levels after both treatments decreased to a lower level at 24 h. These results revealed that expression of the maize *ZmMAG* gene is induced by DNA-damaging agents, and suggest the plant *MAG* gene might function in DNA repair.

## Discussion

More than a century ago, Charles Darwin derived and proposed the concept of the tree of life in *the Origin of Species* based on the hypothesis of gene mutation and vertical inheritance^29^. Since then, one of the goals of biologists is to develop the tree of life to describe the process of evolution^30, 31^. However, subsequent studies revealed that the genetic mechanisms do not only consist of genetic mutation and vertical inheritance, but also includes a novel mechanism, horizontal gene transfer (HGT). HGT plays important roles for the recipient organisms in acquiring new genetic material without the slow process of creating new genes by point mutation and recombination, and in acquiring new genetic material that may control important traits for adaptation to novel environments^32^. HGT, also known as lateral gene transfer (LGT), refers to the movement of genetic material between organisms with reproductive isolation other than *via* vertical inheritance (the transmission of DNA from parent to offspring)^33–35^. It was supposed that HGT is one of the most important forces driving the evolution of prokaryotes and certain unicellular eukaryotes, and leads to the spread of certain adaptive traits, such as antibiotic resistance and virulence^32, 36, 37^. Recent genomic surveys also found horizontally acquired genes in all major lineages of multicellular eukaryotes, including plants, fungi and animals, suggesting that HGT is also crucial for adaptive evolution throughout eukaryotic evolution^38–41^. The ubiquity of horizontally acquired genes in various cellular organisms leads to the concept of “web of life” rather than a progressively branching tree^33, 42^. Thus, detecting the genes acquired through horizontal transfer can not only provide understanding of molecular basis of adaptive evolution for the acceptor organisms, but can also provide references in building the “web of life”.

The biggest environmental change in the history of plant evolution is from aquatic to terrestrial environments. During their transition from water to land, plants had evolved some new phenotypic novelties and metabolic pathways to adapt to the terrestrial habit, including vascular tissues, stomata, cuticles, and the biosynthesis of plant polyamines and hormones^43, 44^. It was suggested that HGT played critically roles in the genomic innovations during the process of adaptive evolution for the colonization of land, such as the development of vascular tissues^45, 46^, the biosynthesis and degradation of some hormones^47, 48^ and disease resistance^49^. From water to terrestrial environments, one of the main environmental differences is that plants are directly exposed to UV and other DNA-damaging agents. Therefore, acquiring the genes that encode proteins for DNA repair is extremely important for the adaptive evolution of land plants.

The protein product of the bacterial *MAG* gene is thought to be involved in the recognition of a variety of base lesions and to initiate their repair *via* the BER pathway^9, 16, 18, 19^. In the present study, we found that the homologs of the *MAG* gene are widely present in streptophytes, from charophyte alga to angiosperm. At least two lines of evidence suggested that the Streptophyta *MAG* gene was acquired through an ancient horizontal gene transfer event from bacteria. First, all the green plant *MAG* genes formed a bootstrap-supported branch in the phylogeny, which is located in the clades of some bacteria such as gammaproteobacteria, deltaproteobacteria, and acidobacteria. Second, the protein products of the Streptophyta *MAG* genes showed the highest similarity to these bacterial homologs in this clade, and importantly, some motifs in the C-terminus are only conserved between land plants and these bacterial MAGs. We also noticed that the homologs of green plant *MAG* gene are present in *Galdieria sulphuraria*, a species of red alga. Because the green plants shared the same ancestor with red algae^50, 51^, the evolutionary scenario that the Streptophyta *MAG* gene was acquired from bacteria through HGT should exclude the possibility that this gene was inherited from the ancestor of green plants and red algae. In the phylogeny, the *G. sulphuraria MAG* gene was not located in the same branch with those in green plants, and more importantly, the protein product of this *G. sulphuraria* gene did not share any specifically evolutionarily conserved motifs with its homologs in streptophytes. Therefore, the most parsimonious explanation is that the origin of the Streptophyta *MAG* gene was the result of an ancient HGT event from bacteria, and the putative donators are gammaproteobacteria, deltaproteobacteria, and acidobacteria.

Charophyte algae, comprising in streptophytes together with land plants, are found in fresh water, with several ranging into brackish habitats, and several groups live in soils, crusts, and other aerial environments^52^. Comparison of the genome sequences with that of green algae and land plants demonstrate that charophyte algae acquired many genes specific to land plants, including those performing the functions in transcription regulation, signal transduction, stress responses, cell wall biogenesis and hormone-related functions^53^. In addition, it was also suggested that the most recent common ancestor of extant land plants and charophyte algae was preadapted for symbiotic associations^54^. In addition to land plants, the *MAG* gene is also present in the genomes of charophyte algae, such as *K. flaccidum* and *P. margaritaceum*. In the present study, we showed that the ancestor of charophyte algae and land plants had acquired the *MAG* gene through an ancient HGT event. In addition, two DNA-damaging agents, including UV radiation and zeocin, were selected to simulate DNA-damaging environments. UV radiation induces two of the most abundant mutagenic and cytotoxic DNA lesions such as cyclobutane–pyrimidine dimers (CPDs) and 6-4 photoproducts (6-4 PPs) and their Dewar valence isomers^9^, while zeocin causes cell death by intercalating into DNA and induces double strand breaks of the DNA for most bacteria, fungi, plant, and animal cells^55^. Our experiments revealed that the expression of the maize *ZmMAG* gene was significantly induced by both UV radiation and zeocin, suggesting that plant *MAG* gene might be involved in the recognition and repair of a variety of base lesions. These results suggested that the ancestor of land plants acquired this gene through an HGT event for the preadaptation to DNA-damaging agents in terrestrial environments.

In general, prokaryotic genes contain no spliceosomal introns. However, nearly all green plant *MAG* genes contain five introns, and their positions and phases are highly conserved across all the lineages of green plants. This result suggested that these introns in Streptophyta *MAG* genes arose through insertions shortly after the HGT event and before the origin of the land plants. The phenomena that horizontally acquired genes further obtained introns has been observed in other HGT genes in plants^44, 45^. Because introns in eukaryotes fulfill a broad spectrum of functions, including virtually every step of mRNA processing, influencing and enhancing gene expression, and maintaining genomic structure^56, 57^, the quick acquisition of introns by the plant *MAG* gene shortly after the HGT event may represent the adaptive evolution to the acceptor genome and new environments. In our analysis, we found that the dominant driving force for *MAG* gene evolution in both streptophytes and bacteria (those in the same clade with plants) was purifying selection, which might contribute to functional stabilization^58^. However, when we used the bacterial genes as a background, positive selection was found to significantly contribute to the evolution of the Streptophyta *MAG* genes. Because point mutations under positive selection is a major force underlying the adaptation of organisms to a new environment^59^, the most reasonable explanation for these results is that the Streptophyta *MAG* gene may have acquired some functional innovations through positive selection shortly after the HGT event but before the separation of major land plant lineages. Although the actual function of the *MAG* gene in higher plants needs further investigation, some studies have also given some clues that the plant *MAG* gene may have gained putative functions other than its homologs in bacteria. For example, the expression of *B. distachyon MAG* gene was found to be regulated by salt stress^26^. In addition, the *Arabidopsis AtMAG* gene was preferentially expressed in meristematic tissue, the developing embryo and endosperm, and organ primordia, and strongly expressed in the mesophyll tissue of the growing leaf, suggesting that it might participate in the progress of cell growth^60^.

## Conclusions

Because there are more DNA-damaging agents in terrestrial environments than in water, the origin and radiation of land plants should acquire new genes with the function of DNA repair. Therefore, understanding the evolutionary pattern of the genes involved in DNA repair will lay the foundation for revealing the genetic mechanism of the origin of land plants. In addition, it can also provide useful information for further genetic improvement of crop plants, because the stratospheric ozone layer, which can protect plant DNA from UV damaging, is continuously experiencing depletion. MAG has been shown to perform the functions in recognizing various base lesions in bacteria, and in initiating the process of the base excision repair for damaged DNA. In the present study, we provide clear evidence that the ancestor of land plants acquired the *MAG* gene through ancient HGT from bacteria. Further investigation revealed that the expression of the plant *MAG* gene is induced by DNA-damaging agents. The Streptophyta *MAG* genes had undergone positive selection during the initial evolutionary period after HGT, which might have led to the *MAG* obtaining functional innovation and fixation in the genome of the land plant ancestor. These findings revealed that the acquisition of *MAG* by the ancestor of land plants through HGT represents a preadaptation to terrestrial environments.

## Materials and Methods

### Sequence data sources

To identify the genes encoding MAG proteins in green plants, the protein sequence of the *Arabidopsis* gene *AtMAG* (AT3G12040)^20, 60^ was used as a query to search the NCBI *nr* and ref_seq protein, the spruce genome project^61^ and Phytozome^62^ databases. Then, the Pfam tool^63^ was used to predict the methylpurine-DNA glycosylase (Pur_DNA_glyco, PF02245) domain. The newly identified MAG sequences detected in green plants were used to search the respective sequence database iteratively. The deduced nucleotide and protein sequences of green plant *MAG* genes identified in this analysis were acquired from the Phytozome and NCBI *nr* and ref_seq databases. The redundant sequences were removed manually according to their chromosomal locations. To detect the *MAG* gene in *Picea glauca*, we searched the NCBI EST database using TBLASTN. Then, UniGene and ORFfinder were used to unite several ESTs into one full-length cDNA.

To identify the homologs of green plant *MAG* genes, BLAST searches against the *nr* protein sequence, NCBI EST and available eukaryotic genome databases were performed using the plant MAG protein sequences as queries. The obtained hits were further analyzed through a Pfam search to confirm the presence of the Pur_DNA_glyco domain. Protein sequences were sampled for a further combined phylogenetic analysis from representative groups within each domain of life (bacteria, archaea and eukaryotes) based on the BLASTP results.

### Phylogenetic tree reconstruction

Multiple alignments for all the selected representative protein sequences were performed with Clustal X^64^. The gaps and ambiguously aligned sites were removed manually. Phylogenetic analyses were performed using maximum likelihood (ML) and neighbor-joining (NJ) methods using PhyML v3.0^65^ and MEGA v7.0^66^, respectively. The program ModelGenerator^67^ was used to identify the optimal model of protein substitution and rate heterogeneity. The ML phylogenetic analyses were conducted with the following parameters: Dayhoff model, estimated proportion of invariable sites, 4 rate categories, estimated gamma distribution parameter, and optimized starting BIONJ tree. The Jones-Taylor-Thornton (JTT) model was employed for the construction of NJ trees. A total of 100 non-parametric bootstrap samplings were carried out to estimate the support level for each internal branch for both the ML and NJ trees. The branch lengths and topologies of all phylogenies were calculated with PhyML.

### Detection of selective constrains

To test the selective constrains of the *MAG* genes during the long period of evolution in both green plants and some selected bacteria, the values of the ${d}_{N}/{d}_{S}$ ratio (*ω*) for two groups of *MAG* genes were calculated with the program *codeml* in PAML v4.9^68^. In this analysis, only bacterial *MAG* genes located in the same branch as green plant homologs were used. The PAL2NAL program^69^ was utilized for conversion of multiple alignment of proteins into the corresponding codon-based nucleotide alignment, which, in turn, was input into the *codeml* program in PAML. Three likelihood ratio tests (LRTs), including M0 *vs*. M3, M1a *vs*. M2a, and M7 *vs*. M8, were employed to examine the selective pressure. The LRT of the M0 *vs*. M3 comparison was used to test the heterogeneity in *ω* among the codon sites, while the other two LRTs were used to detect the role of positive selection. For one LRT, twice the difference of the log likelihood of the two models was compared with chi-square (*χ* ^2^) statistics, with degrees of freedom (DFs) equal to the difference in the number of parameters. In our analyses, the DFs were 3 for the M0/M3 LRT and 2 for the M1a/M2a and M7/M8 LRTs, respectively^70, 71^.

The improved branch-site model^27^ was also used to detect the effect of positive selection acting on the Streptophyta *MAG* genes following HGT. In these tests, we compared the null model (*ω* fixed to 1) with the alternative model (free *ω*). The branch of streptophytes was used as the foreground, while that containing the genes from bacteria, the putative donor of the Streptophyta *MAG* gene, was used as the background. Here, only the bacterial genes falling within the same branch as the Streptophyta genes were used. The Bayes empirical Bayes procedure^28^ in *codeml* was used to calculate the posterior probability that each site was subject to positive selection in the foreground branch.

### Plant material, growth and stress treatments

Seeds of maize (*Zea mays* L.) inbred line B73 were surface-sterilized for 15 min in 0.05% sodium hypochlorite, and then washed thoroughly with sterile water. The sterilized seeds were germinated on filter paper saturated with distilled water in darkness for 2 days at 28 °C. For UV treatment, the uniformly germinated seeds were grown with sieved topsoil in the growth chamber, with a photoperiod of 14/10 h at 28/25 °C (light/night), 1200 μmol photons m^−2^ s^−1^ photosynthetically active radiation, and 70% relative humidity. Plants received 100 ml of water every day for 2 weeks. Two-week-old seedlings were moved to the UV light with 10 kJ m^−2^ d^−1^. The leaves of the seedlings were sampled at 0, 3, 6, 12, 24, 48 h after UV treatment, frozen in liquid nitrogen and stored at −80 °C for further analysis. For zeocin treatment, the uniformly germinated seeds were grown with culture solution in the growth chamber. The growth condition is the same as described above. Two-week-old seedlings were transferred to culture solutions containing 100 μM zeocin. The whole seedlings were sampled at 0, 3, 6, 12, 24, 48 h after zeocin treatment, frozen in liquid nitrogen and stored at −80 °C for further analysis.

### RNA isolation and real-time PCR

Total RNA was extracted from frozen tissue using RNAsimple Total RNA Kit (TIANGEN) according to the manufacturer’s instructions. To check the *ZmMAG* expression level in maize plants in response to UV and zeocin treatment, approximately 2 μg of total RNA from each sample was used for first-strand cDNA synthesis by 5X All-In-One RT MasterMix (abm). Quantitative real-time PCR was performed with the EvaGreen 2X qPCR MasterMix (abm) with the following reaction conditions: 95 °C for 10 mins, then 30 cycles of 95 °C for 15 secs, 60 °C for 60 secs. The constitutively expressed *ZmActin* gene (GenBank accession no. J01238) was used as an internal control to normalize data. Quantitative gene expression was analyzed by comparative CT (Delta–Delta CT) method^72^. The primers were 5′-TTACGGGACCAGGAAAGGTTGG-3′ and 5′-AGGATGGTTCGACCAGTCAGTG-3′ for the *ZmMAG* gene, and 5′-GATGATGCGCCAAGAGCTG-3′ and 5′-GCCTCATCACCTACGTAGGCAT-3′ for *ZmActin* gene. All experiments were carried out using three biological replicates. The *ZmMAG* expression data were expressed as means ± standard deviation (SD). Statistical analysis was performed by one-way ANOVA and Tukey’s honestly significant difference test for significance (*P* < 0.05) by using IBM SPSS software (Version 22).

## Electronic supplementary material

Supplementary Information
