# Supplementary material for: Ancestor of land plants acquired the DNA-3-methyladenine glycosylase (MAG) gene from bacteria through horizontal gene transfer
Source: Sci Rep. 2017 Aug 24;7:9324. doi: 10.1038/s41598-017-05066-w (PMC5570899; doi:10.1038/s41598-017-05066-w)
Supplement: Supplementary file 1 — Supplementary Information [file 41598_2017_5066_MOESM1_ESM.pdf]

Supplementary Tables and Figures for:

Ancestor of land plants acquired the DNA-3-methyladenine glycosylase (*MAG*) gene from bacteria through horizontal gene transfer

Huimin Fang[1, #], Liexiang Huangfu[1, #], Rujia Chen[1], Pengcheng Li[1], Shuhui Xu[1], Enying Zhang[1, 2], Wei Cao[1], Li Liu[1], Youli Yao[1], Guohua Liang[1], Chenwu Xu[1,*], Yong Zhou[1,*], Zefeng Yang[1,*]

[1] Jiangsu Key Laboratory of Crop Genetics and Physiology/Co-Innovation Center for Modern Production Technology of Grain Crops, Key Laboratory of Plant Functional Genomics of the Ministry of Education, Yangzhou University, Yangzhou, 225009, China

[2] College of Agronomy and Plant Protection, Qingdao Agricultural University, 266109, Qingdao, China

# These authors contributed equally to this work.

Corresponding author: Zefeng Yang

Email: zfyang@yzu.edu.cn

Chenwu Xu

Email: qtls@yzu.edu.cn

Yong Zhou

Email: zhouyong@yzu.edu.cn

Supplementary Table S1. List of 52 green plant *MAG* genes in 50 representative genomes.

No.	Lineage	Species	Gene	Locus_Gene	Locus_Protein	Protein length	Intron	Chr/scaffold	Location
1	Monocots	*Zea mays*	*ZmMAG*	LOC100285435	NP_001151800	263	5	5	211489338..211492095
2	Monocots	*Sorghum bicolor*	*SbMAG*	SORBIDRAFT_04g034700	XP_002454640	263	5	4	64529095..64531120
3	Monocots	*Setaria italica*	*SiMAG*	LOC101765866	XP_004954086	292	5	1	39452008..39454395
4	Monocots	*Oryza sativa*	*OsMAG*	LOC4330890	XP_015624253	286	5	2	32695461..32697585
5	Monocots	*Phoenix dactylifera*	*PdMAG*	LOC103706763	XP_008789196	259	5	NW_008246560	255029..259853
6	Monocots	*Brachypodium distachyon*	*BdMAG*	LOC100822861	XP_003570532	303	5	3	57054909..57057388
7	Monocots	*Musa acuminata subsp. malaccensis*	*MaMAG*	LOC103992180	XP_009410066	247	5	7	21346611..21350081
8	Monocots	*Elaeis guineensis*	*EgMAG*	LOC105053127	XP_010932474	259	5	10	24327110..24332019
9	Eudicots	*Arabidopsis thaliana*	*AtMAG*	AT3G12040	NP_187811	254	5	3	3835129..3836833
10	Eudicots	*Ricinus communis*	*RcMAG*	LOC8263220	XP_002530360	258	5	NW_002994704	83340..87289
11	Eudicots	*Arabidopsis lyrata subsp. lyrata*	*AlMAG*	ARALYDRAFT_478556	XP_002884879	253	5	NW_003302553	4866813..4865295
12	Eudicots	*Glycine max*	*GmMAG*	LOC100813424	XP_003554221	276	5	19	40968673..40972183
13	Eudicots	*Medicago truncatula*	*MtMAG*	MTR_7g093540	XP_003625290	271	5	7	37190517..37194062
14	Eudicots	*Vitis vinifera*	*VvMAG*	LOC100250307	XP_002280176	277	5	8	20388979..20393360
15	Eudicots	*Cucumis sativus*	*CsMAG*	LOC101219786	XP_004136560	281	5	3	30790625..30793964
16	Eudicots	*Solanum*	*SlMAG*	LOC101264772	XP_004249291	288	5	10	61799679..61804202

		lycopersicum							
17	Eudicots	*Solanum tuberosum*	*StMAG*	LOC102599279	XP_006351353	288	5	NW_006239057	1263265..1266654
18	Eudicots	*Capsella rubella*	*CrMAG*	CARUB_v10014465mg	XP_006298394	252	5	NW_006238921	3928063-3926047
19	Eudicots	*Populus trichocarpa*	*PtMAG*	POPTR_0016s06000g	XP_002322736	260	5	XVI	3924106..3927343
20	Eudicots	*Eutrema salsugineum*	*EsMAG*	EUTSA_v10021378mg	XP_006407378	252	5	NW_006256885	5503168-5504870
21	Eudicots	*Citrus clementina*	*CcMAG*	CICLE_v10012466mg	XP_006429905	269	5	NW_006262139	19082593-19085479
22	Eudicots	*Citrus sinensis*	*CiMAG*	LOC102620793	XP_006492840	269	5	NW_006257094	204803..207970
23	Eudicots	*Theobroma cacao*	*TcMAG*	TCM_024945	XP_007029030	309	5	5	29699395..29702862
24	Eudicots	*Phaseolus vulgaris*	*PvMAG*	PHAVU_001G143900g	XP_007162344	286	5	1	39385867..39388690
25	Eudicots	*Prunus persica*	*PpMAG*	PRUPE_ppa009882mg	XP_007204708	273	5	NW_006760201	11188517-11185794
26	Eudicots	*Prunus mume*	*PmMAG*	LOC103339397	XP_008240907	273	5	8	6490118..6493222
27	Eudicots	*Cucumis melo*	*CmMAG*	LOC103486748	XP_008443030	282	5	NW_007546278	3491158..3494678
28	Eudicots	*Brassica rapa*	*BrMAG*	LOC103870283	XP_009146652	253	5	A5	21415704..21417286
29	Eudicots	*Eucalyptus grandis*	*EuMAG*	LOC104425501	XP_010036500	302	5	NW_010092448	23114307..23118028
30	Eudicots	*Morus notabilis*	*MnMAG*	L484_028033	XP_010111376	255	5	NW_010367622	2649262-2652060
31	Eudicots	*Camelina sativa*	*ClMAG1*	LOC104776133	XP_010498453	259	5	1	4926069..4928002
32	Eudicots	*Camelina sativa*	*ClMAG2*	LOC104745418	XP_010464954	259	5	15	5206114..5209118
33	Eudicots	*Camelina sativa*	*ClMAG3*	LOC104764965	XP_010486887	260	5	19	5491373..5493278
34	Eudicots	*Tarenaya hassleriana*	*ThMAG*	LOC104809936	XP_010534365	257	5	NW_010965122	1308687..1311345
35	Eudicots	*Beta vulgaris subsp. vulgaris*	*BvMAG*	LOC104889174	XP_010672632	280	5	3	17730543..17733724

36	Eudicots	*Populus euphratica*	*PeMAG*	LOC105136027	XP_011039480	260	5	NW_011499931	323807..326636
37	Eudicots	*Sesamum indicum*	*SeMAG*	LOC105159663	XP_011075101	286	5	4	348954..352256
38	Eudicots	*Fragaria vesca subsp. vesca*	*FvMAG*	LOC101310624	XP_011467123	261	5	6	18606192..18609073
39	Eudicots	*Gossypium raimondii*	*GrMAG*	LOC105780867	XP_012460848	260	5	12	21075654..21078683
40	Eudicots	*Erythranthe guttata*	*ErMAG*	LOC105955243	XP_012834409	290	5	NW_012193095	358492..360174
41	Eudicots	*Brassica oleracea var. oleracea*	*BoMAG*	LOC106293067	XP_013584197	261	5	C5	41863462..41865079
42	Eudicots	*Brassica napus*	*BnMAG*	LOC106413976	XP_013710148	250	5	C8	7827853..7829436
43	Eudicots	*Vigna radiata var. radiata*	*VrMAG*	LOC106757343	XP_014495472	278	5	3	420657..423273
44	Eudicots	*Ziziphus jujuba*	*ZjMAG*	LOC107409860	XP_015872770	261	5	NW_015457595	7..1682
45	Eudicots	*Arachis duranensis*	*AdMAG*	LOC107492878	XP_015969421	260	5	A06	12249492..12253285
46	Eudicots	*Arachis ipaensis*	*AiMAG*	LOC107645400	XP_016204897	244	5	B06	3470380..3473124
47	Basal angiosperm	*Amborella trichopoda*	*AmMAG*	LOC18439318	XP_006849548	268	5	NW_006499705	2794866..2801692
48	Gymnosperm	*Picea glauca* [b]	*PgMAG*	EST: EX431593, EX365126, EX365487, DR594722	/	/	/	/	/
49	Pteridophyta	*Selaginella moellendorffii* [a]	*SmMAG*	fgenesh1_pm.C_scaffold_21000058	232080	238	5	scaffold_21	1705132..1706138
50	Liverworts	*Marchantia polymorpha subsp. polymorpha*	*MpMAG*	AXG93_4875s1290	OAE34409	377		LVLJ01000408	408192..409622

51	Mosses	*Sphagnum fallax* [a]	*SfMAG*	Sphfalx0146s0034	/	335	7	super_146	505066..508685
52	Charophyta	*Klebsormidium flaccidum*	*KmMAG*	KFL_001180170	GAQ82638	263	5	DF237067	79105..81563

Note: a, these sequences were acquired from Phytozome; b, the *MAG* gene in *Picea glauca* was predicted using four high similarity ESTs hits. All other sequences were obtained from NCBI.

Supplementary Table S2 Summary of statistics for detecting selective constraints using site-specific models.

Lineage	Model	p	ln*L*	Estimate of parameters	*df*	$2\Delta L$	Positively selected sites
Bacteria	M0: one-ratio	1	-3025.0000	$\omega = 0.0486$	/	/	/
	M1a: nearly neutral	2	-2975.6316	$p_0 = 0.8024, p_1 = 0.1975$<br>$\omega_0 = 0.0291, \omega_1 = 1.0000$	/	/	/
	M2a: positive selection	4	-2975.6316	$p_0 = 0.8024, p_1 = 0.1780, p_2 = 0.0195$<br>$\omega_0 = 0.0291, \omega_1 = 1.0000, \omega_2 = 1.0000$	2	0	NAN
	M3: discrete	5	-2948.2265	$p_0 = 0.2905, p_1 = 0.5551, p_2 = 0.1544$<br>$\omega_0 = 0.0000, \omega_1 = 0.0411, \omega_2 = 0.4173$	4	154.5470**	NAN
	M7: β	2	-2954.0248	$p = 0.4655, q = 5.4813$	/	/	/
	M8: $\beta \& \omega$	4	-2952.8029	$p_0 = 0.9295, p = 0.9294, q = 12.3371$<br>$p_1 = 0.0705, \omega = 1.0000$	2	2.4438	NAN
Streptophytes	M0: one-ratio	1	-11627.0359	$\omega = 0.0740$	/	/	/
	M1a: nearly neutral	2	-11464.3325	$p_0 = 0.9222, p_1 = 0.0778$<br>$\omega_0 = 0.0565, \omega_1 = 1.0000$	/	/	/
	M2a: positive selection	4	-11464.3325	$p_0 = 0.9222, p_1 = 0.0610, p_2 = 0.0168$<br>$\omega_0 = 0.0565, \omega_1 = 1.0000, \omega_2 = 1.0000$	2	0	NAN
	M3: discrete	5	-11250.0018	$p_0 = 0.5612, p_1 = 0.3438, p_2 = 0.0950$<br>$\omega_0 = 0.0111, \omega_1 = 0.1063, \omega_2 = 0.4088$	4	754.0682**	NAN

	M7: β	2	-11249.4030	$p = 0.3693, q = 3.3986$	/	/	/
	M8: $\beta \& \omega$	4	-11249.2514	$p_0 = 0.9246, p = 0.3805, q = 3.6787$<br>$p_1 = 0.0054, \omega = 1.0000$	2	0.3032	NAN

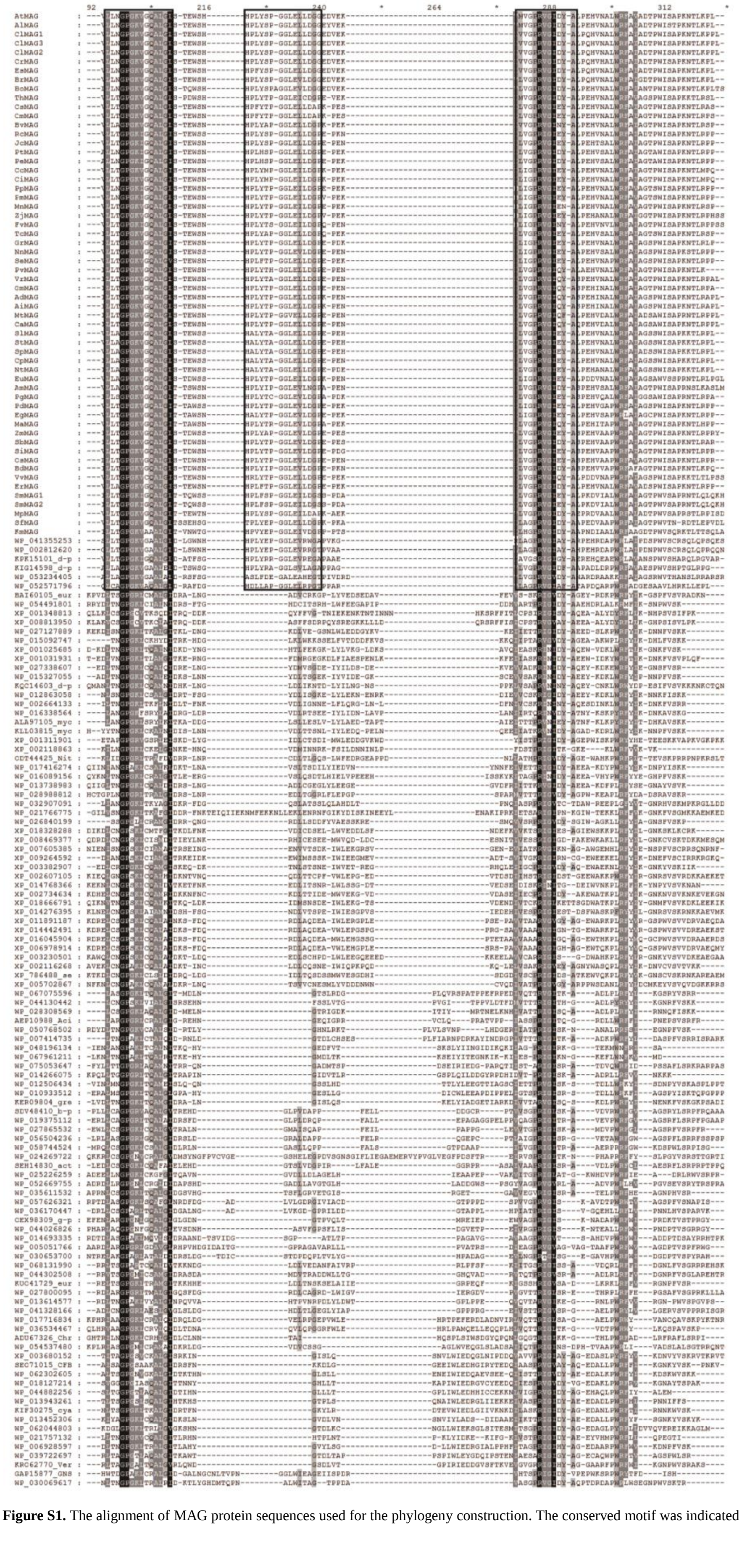

Supplementary Figure S1. The alignment of MAG protein sequences used for the phylogeny construction. The conserved motif was indicated by a box.

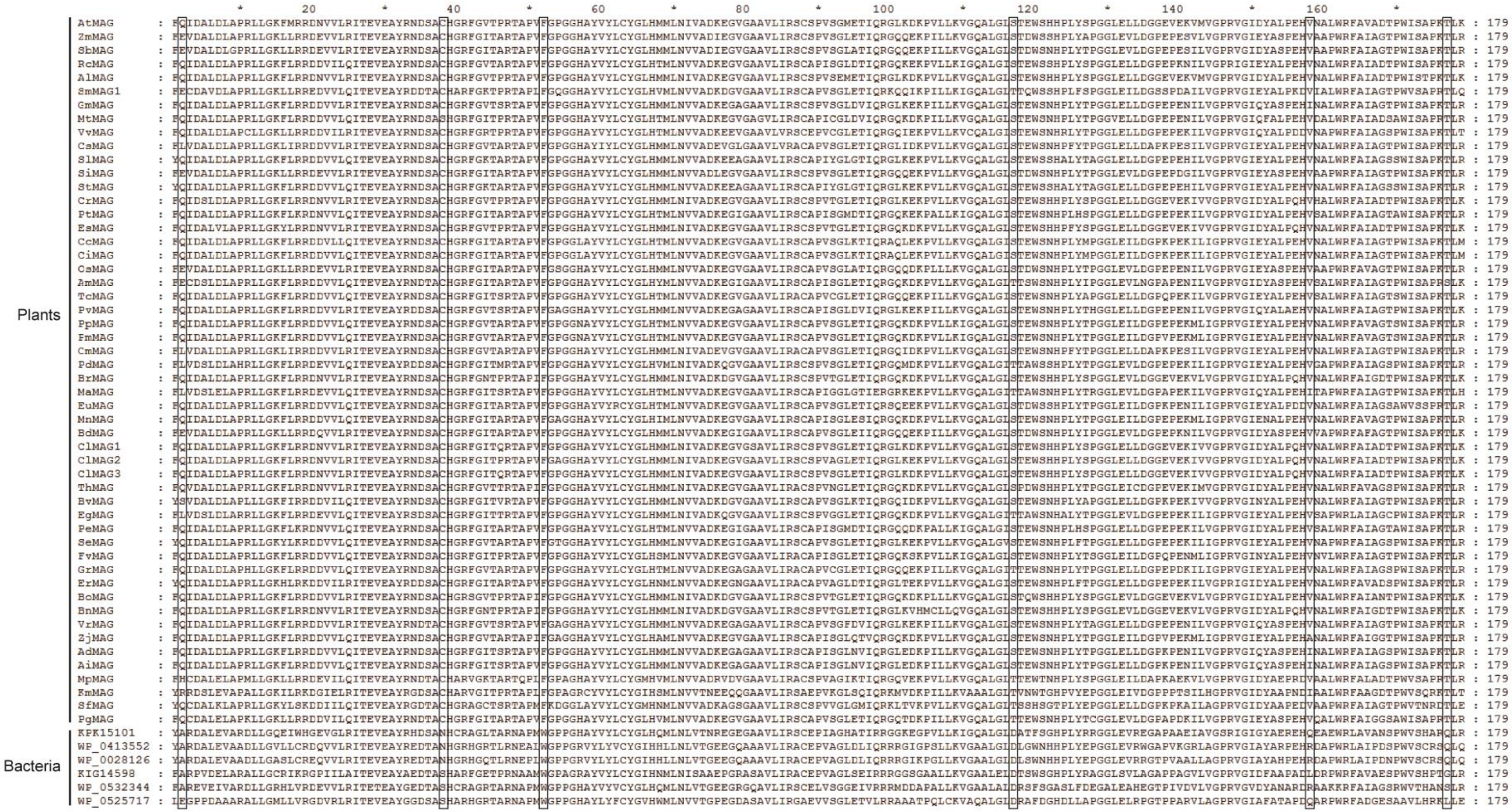

Supplementary Figure S2. The alignment of the selected MAG protein sequences in green plants and selected bacteria, showing the positively selected sites in green plants.
